# LncRNA NCK1-AS1 in plasma distinguishes oral ulcer from early-stage oral squamous cell carcinoma

**DOI:** 10.1186/s40709-020-00126-1

**Published:** 2020-11-11

**Authors:** Fei Le, Yangqian Ou, Ping Luo, Xiaoming Zhong

**Affiliations:** 1 Department of Head and Neck Surgery, Jiangxi Province Tumor Hospital, Nanchang City, Jiangxi Province 330029 People’s Republic of China; 2 Department of Intensive Medicine, Jiangxi Province Tumor Hospital, Nanchang City, Jiangxi Province 330029 People’s Republic of China; 3 Department of Surgical Oncology, Nanchang Third Hospital, Nanchang City, Jiangxi Province 330002 People’s Republic of China; 4 Department of Radiotherapy, Jiangxi Province Tumor Hospital, No.519 Beijing East Road, Nanchang City, Jiangxi Province 330029 People’s Republic of China

**Keywords:** Oral squamous cell carcinoma, Oral ulcer, lncRNA NCK1-AS1, miR-100

## Abstract

**Background:**

Oral squamous cell carcinoma (OSCC) at early stages can be misdiagnosed as an oral ulcer (OU) due to similar symptoms, such as chronic and indurated ulcer. LncRNA NCK1-AS1 has been characterized as a key player in cervical cancer, while its role in OSCC is unknown.

**Methods:**

All participants were selected at Jiangxi Province Tumor Hospital from December 2016 to December 2018. Expression levels of NCK1-AS1 and miR-100 in plasma from both OSCC and OU patients were measured by RT-qPCR. Diagnostic analysis was performed through ROC curve. Potential interactions between NCK1-AS1 and miR-100 were detected by cell transfection experiments. Cell invasion and migration were assessed by Transwell assays.

**Results:**

The expression of NCK1-AS1 was upregulated in early-stage OSCC patients but not in OU patients. Upregulation of NCK1-AS1 distinguished OSCC patients from OU patients. The expression of miR-100 was inversely correlated with the expression of NCK1-AS1. Overexpression of NCK1-AS1 was followed by promoted OSCC cell invasion and migration. Overexpression of miR-100 did not affect the expression of NCK1-AS1 but inhibited the role of NCK1-AS1.

**Conclusions:**

Therefore, NCK1-AS1 may promote the metastasis of OSCC by downregulating miR-100.

## Background

Oral squamous cell carcinoma (OSCC) accounts for more than 80% of head and neck cancer, which is a common malignancy in clinical practices [1, 2]. Smoking, alcohol abuse, betel quid chewing consumption and HPV infections have been identified as the major risk factors for OSCC [3]. However, the molecular mechanism of OSCC pathogenesis remains unclear. Although efforts have been made to develop an effective treatment of OSCC, mortality rate of OSCC patients is still high, with an overall 5 year survival rate lower than 50% [4]. In addition, OSCC can be easily diagnosed as other oral lesions [5], leading to delayed treatment. Therefore, novel therapeutic targets and accurate diagnostic markers are urgently needed.

The tumorigenesis and progression of OSCC are complicated and involve multiple genetic processes [6, 7]. The development of OSCC may provide new insights into its treatment and prevention. Long non-coding RNAs (lncRNAs, > 200 nt) are frequently dysregulated in cancer and may regulate cancer-related gene expression [8, 9]. Therefore, lncRNAs are promising targets for the development of anti-cancer approaches [10]. However, characterization of lncRNAs is required before their clinical applications. A recent study reported that lncRNA NCK1-AS1 is a key player in the development of chemoresistance in cervical cancer cells to cisplatin [11]. We observed upregulated NCK1-AS1 in plasma of OSCC patients from our preliminary deep sequencing data (data not shown). Therefore, this study was carried out to investigate the role of NCK1-AS1 in OSCC.

## Methods

### Research subjects

Research subjects included 55 OSCC patients (29 males and 26 females, age range 35 to 66 years old, mean age 49.8 ± 6.4 years old), 49 OU patients (26 males and 23 females, age range 34 to 67 years old, mean age 49.3 ± 5.4 years old), and 55 healthy participants (39 males and 26 females, age range 33 to 68 years old, mean age 50.1 ± 5.6 years old). Clinical data of 55 OSCC were presented in Table 1. The sizes of oral OU in 49 OU patients ranged from 0.5 to 1.3 cm. Lesions were local and found on any area of oral mucosa. All participants were selected at Jiangxi Province Tumor Hospital from December 2016 to December 2018. Inclusion criteria of OSCC and UC patients were: (1) new cases; (2) no initiated therapy; (3) for OSCC patients, clinical stages should be I (n = 18) or II (n = 37), which are early stages. Exclusion criteria were: (1) other clinical disorders; (2) family history of malignancies; (3) previous history of malignancies. Healthy participants were enrolled to match the age and gender distributions of patient groups. All participants signed the informed consent. This study was approved by the Ethics Committee of Jiangxi Province Tumor Hospital.

**Table 1:** Correlation between plasma levels of NCK1-AS1 and patients’ clinical data

Variables		High (n = 27)	Low (n = 28)	Chi square	*p*
Gender					
Male	29	12	17	0.09	0.79
Female	26	15	11		
Age (years)					
> 50	27	15	12	0.89	0.35
< = 50	28	12	16		
Location					
Lower	12	5	7	1.47	0.48
Middle	22	13	9		
Upper	21	9	12		
Tumor size					
2–4 cm	18	11	7	1.55	0.21
< 4 cm	37	16	21		
TNM stage					
T1N0m0	18	11	7	1.55	0.21
T2N0m0	37	16	21		

### Plasma and cells

Prior to therapy, fasting blood (5 ml) was collected from each participant. Blood samples were transferred to EDTA tubes and were centrifuged at 1200 × *g* for 12 min to collect supernatant, and plasma samples were prepared. OSCC cell lines SCC090 and SCC25 (ATCC, USA) were used. A mixture of 10% FBS and 90% EMEM was used to cultivate cells at 37 °C with 5% CO_2_.

### Cell transfections

The NCK1-AS1 expression vector was constructed using pcDNA3 vector obtained from Sangon (Shanghai, China). Mimic of miR-100 and negative control (NC) miRNA, as well as NCK1-AS1 siRNA and NC siRNA were purchased from Sigma-Aldrich (USA). SCC090 or SCC25 cells (10^5^) were transfected with 10 nM vector, 40 nM siRNA or 40 nM miRNA using lipofectamine 2000 reagent (Invitrogen, USA). Cells without transfections were used to serve as the control group (C). Empty vector-, siRNA- or NC miRNA-transfected cells were NC cells. Cells were collected at 24 h after transfection to perform all subsequent experiments.

### Real-time quantitative PCR

SCC25 and SCC090 cells (2 × 10^5^ cells collected at 24 h post-transfection) or 0.15 ml plasma was mixed with 1 ml Trizol reagent (Invitrogen, USA) to extract total RNAs. Following DNase I digestion, SensiFAST™ cDNA Synthesis Kit (Bioline, USA) and KAPA SYBR® FAST Universal Kit (Sigma-Aldrich, USA) were used to perform reverse transcription and prepare qPCR reaction mixtures. The expression of NCK1-AS1 was detected with 18S rRNA as the internal control. The miRNA Isolation Kit (RMI050, Geneaid, USA) was used to extract miRNA from SCC25 and SCC090 cells (2 × 10^5^ cells collected at 24 h post-transfection) or 0.15 ml plasma. MystiCq® microRNA cDNA Synthesis Mix (Sigma-Aldrich, USA) was used to synthesize cDNA and qPCR reactions were carried out using miScript SYBR Green PCR Kit (QIAGEN, Shanghai, China). The expression of miR-100 was determined with U6 as endogenous control. Primer sequences were 5′-AGTTCAGCCCCCACTGCTCT-3′ (forward) and 5′-TGGTTTGAGTTCCCATTTCTC-3′ (reverse) for NCK1-AS1; 5′-TACCACATCCAAGGAAGCA-3′ (forward) and 5′-TTTTTCGTCACTACCTCCCC-3′ (reverse) for 18S rRNA; 5′-ATATGGAACGCTTCACGAATTT-3′ (forward) and 5′-TCGCTTCGGCAGCACATATAC-3′ (reverse) for U6. The forward primer of miR-100 was 5′-AACCCGUAGAUCCGAACUUG-3′. Poly (T) was used as the reverse primer of miR-100. Each experiment included 3 replicates. All Ct values were calculated based on the 2^−ΔΔCT^ method.

### Methylation-specific PCR (MSP)

Genomic DNA Extraction Kit (ab156900, Abcam, UK) was used to extract genomic DNA from SCC25 and SCC090 cells at 24 h post-transfection. DNA was converted using the EZ DNA Methylation-Gold™ kit (ZYMO Research, USA) and DNA methylation was detected using 2X Taq FroggaMix (FroggaBio, Canada) to perform routine PCR reactions. MSP primers and non-MSP primers were used to amplify full length methylated and un-methylated miR-100 precursor, respectively. MSP primers were: 5′-CCTGTTGCCACAAACCCGTAGATC-3′ (forward) and 5′-CCTAACAGACACATACCTATAG-3′ (reverse). Non-MSP primers were: 5′-TTTGTTGTTATAAATTCGTAGATT-3′ (forward) and 5′-CCTAACAAACACATACCTATAA-3′ (reverse).

### Cell migration and invasion assay

The invasion and migration of SCC25 and SCC090 cells were assessed using Transweel inserts (8 μm, Corning, USA). A total of 6000 cells in 0.1 ml serum-free medium were transferred to the upper chamber, and the lower chamber was filled with medium containing 20% FBS. Matrigel (Millipore, USA)-coated and uncoated membranes were used for invasion and migration assays, respectively. Cells were incubated at 37 °C for 12 h, and 1% crystal violet (Sigma-Aldrich, USA) was used to stain the lower surface at 25 °C for 20 min. An optical microscope was used to observe the stained cells.

### Statistical analysis

Data were expressed as mean values of 3 biological replicates. ANOVA Tukey’s test was used to compare differences among multiple groups. Linear regression was used to analyze correlations. The 55 patients were divided into high (n = 27) and low (n = 28) plasma NCK1-AS1 level groups with the median level as a cutoff value. Chi-squared test was used to analyze the correlation between the expression levels of NCK1-AS1 and patients’ clinical data. ROC curve was used for diagnostic analysis. *p* < 0.05 was statistically significant.

## Results

### The expression of NCK1-AS1 was upregulated in early-stage OSCC patients but not in OU patients

**Fig. 1:**
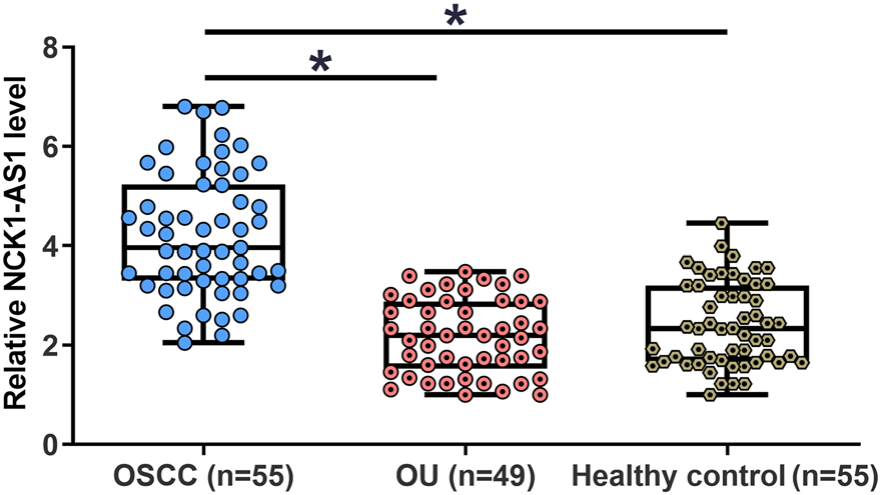
The expression of NCK1-AS1 was upregulated in early-stage OSCC patients but not in OU patients. The expression of NCK1-AS1 in OSCC patients (n = 55), OU patients (n = 49) and healthy participants (n = 55) were detected by qPCR and data were compared by performing one-way ANOVA and Tukey test. It was observed that the expression levels of NCK1-AS1 were significantly higher in OSCC group in comparison to that in OU and healthy participant groups. In contrast, the expression levels of NCK1-AS1 were not significantly different between OU and healthy participant groups.*p < 0.05

The expression of NCK1-AS1 in OSCC patients (n = 55), OU patients (n = 49) and healthy participants (n = 55) were detected by qPCR. The expression levels of NCK1-AS1 in plasma were significantly higher in OSCC group in comparison to that in OU and healthy participant groups (Fig. 1, *p* < 0.05). In contrast, no significant differences in the expression levels of plasma NCK1-AS1 were found between OU and control groups (Fig. 1). Chi-squared test revealed no significant correlations between the expression levels of NCK1-AS1 and patients’ gender, age, tumor size, tumor location and tumor TNM stage.

### High expression levels of NCK1-AS1 showed diagnostic value for OSCC

**Fig. 2:**
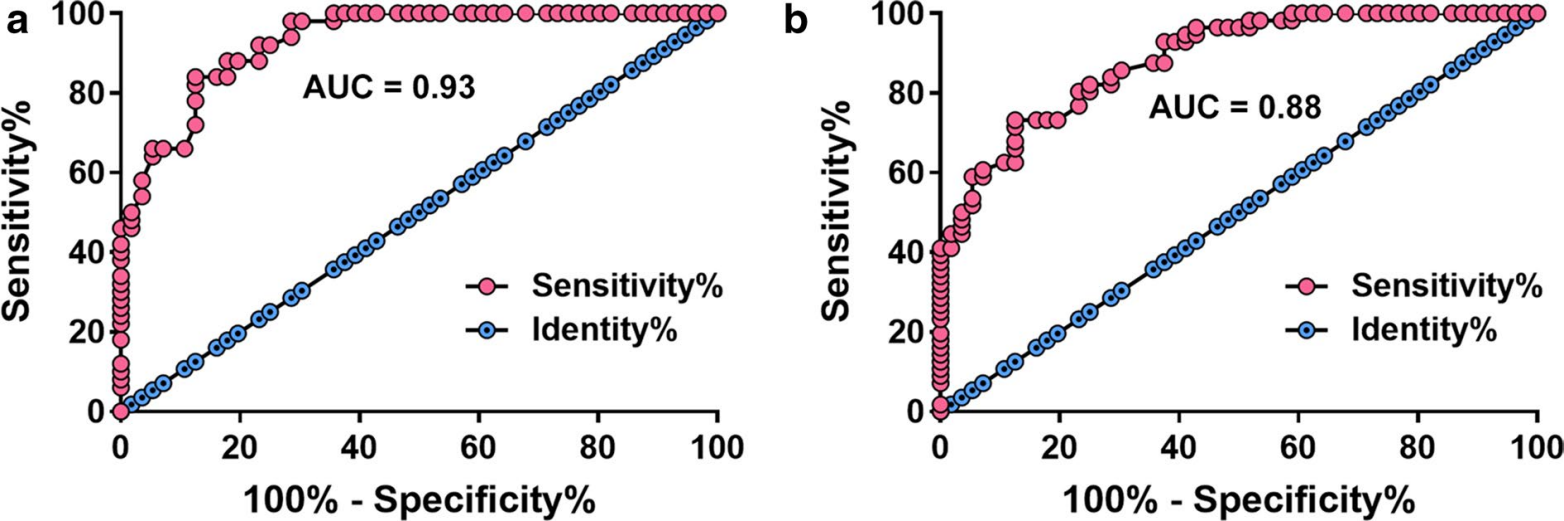
High expression levels of NCK1-AS1 distinguished OSCC patients from OU patients and healthy participants. ROC curve analysis was performed with the 55 early-stage OSCC patients as true positive cases and OU patients or healthy participants as true negative cases. It was observed that upregulated plasma levels of NCK1-AS1 distinguished OSCC patients from OU patients (**a**) and healthy participants (**b**)

In ROC analysis, true positive cases were the 55 early-stage OSCC patients. When true negative cases were OU patients, area under the curve (AUC) was 0.93, with standard error of 0.023 and 95% confidence interval of 0.88–0.97 (Fig. 2a). With healthy participants with negative controls, AUC was 0.88, with standard error of 0.030 and 95% confidence interval of 0.83–0.94 (Fig. 2b).

### The expression of miR-100 was inversely correlated with the expression of NCK1-AS1

**Fig. 3:**
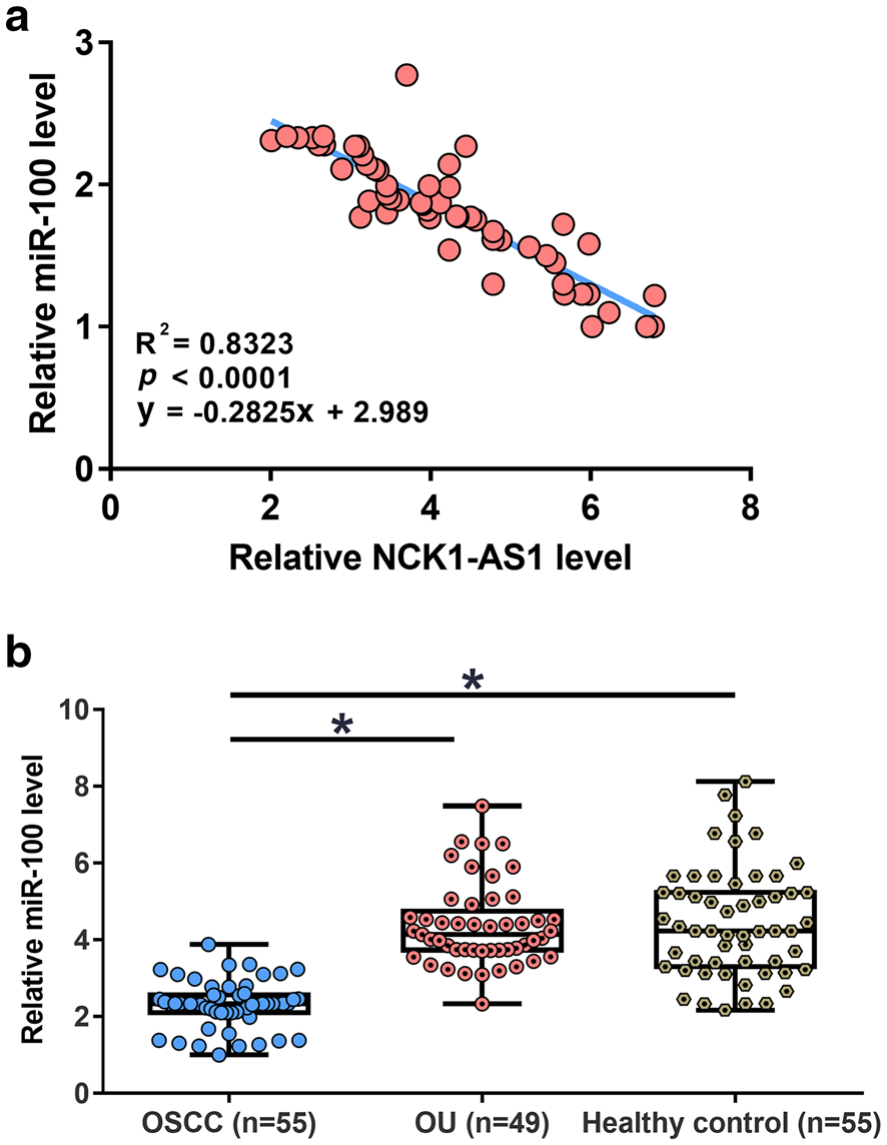
The expression of miR-100 was inversely correlated with NCK1-AS1. ROC curve analysis showed that plasma miR-100 and NCK1-AS1 were inversely and significantly correlated in OSCC patients (**a**). The expression of miR-100 in 55 early-stage OSCC patients, 49 OU patients, and 55 healthy participants were detected by RT-qPCR. It was observed that plasma levels of miR-100 were significantly lower in OSCC patients in comparison to that in OU patients and healthy participants (**b**). *p < 0.05

Correlations between plasma miR-100 and NCK1-AS1 across OSCC samples were analyzed. The expression of miR-100 and NCK1-AS1 were inversely and significantly correlated in OSCC patients (Fig. 3a). The expression of miR-100 in 55 early-stage OSCC patients, 49 OU patients, and 55 healthy participants were detected by RT-qPCR. The results showed that the expression levels of miR-100 were significantly lower in OSCC patients in comparison to that in OU patients and healthy participants (Fig. 3b).

### NCK1-AS1 downregulated miR-100 in OSCC cells

**Fig. 4:**
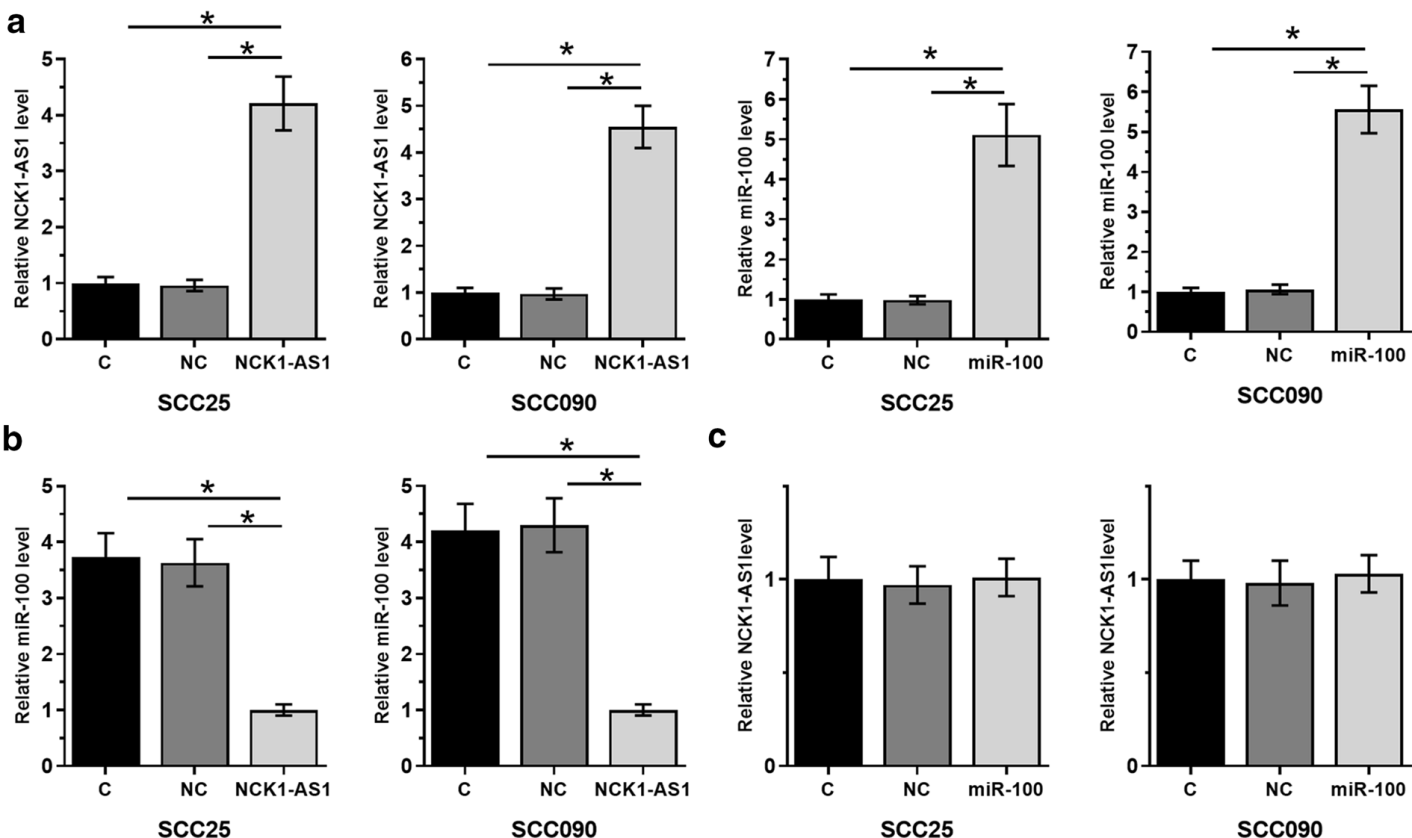
Overexpression of NCK1-AS1 resulted in the downregulation of miR-100 in OSCC cells. In comparison to C and NC groups, the expression levels of NCK1-AS1 and miR-100 were significantly increased at 24 h after transfections (**a**), indicating successful transfections. In addition, cells with the overexpression of NCK1-AS1 showed significantly downregulated miR-100 (**b**). However, cells with the overexpression of miR-100 showed no significantly altered expression of NCK1-AS1 (C). *p < 0.05

SCC25 and SCC090 cells were transfected with either NCK1-AS1 expression vector or the mimic of miR-100. Overexpression of NCK1-AS1 and miR-100 was confirmed at 24 h after transfections (Fig. 4a, *p* < 0.05). In addition, cells with NCK1-AS1 expression showed significantly downregulated miR-100 (Fig. 4b, *p* < 0.05). In contrast, overexpression of miR-100 did not alter the expression of NCK1-AS1 (Fig. 4c). Silencing of NCK1-AS1 was also achieved in both SCC25 and SCC090 cells (Additional file 1. Figure S1a). It was observed that silencing of NCK1-AS1 resulted in the upregulation of miR-100 (Additional file 1. Figure S1b).

### NCK1-AS1 promoted OSCC cell migration and invasion through miR-100

**Fig. 5:**
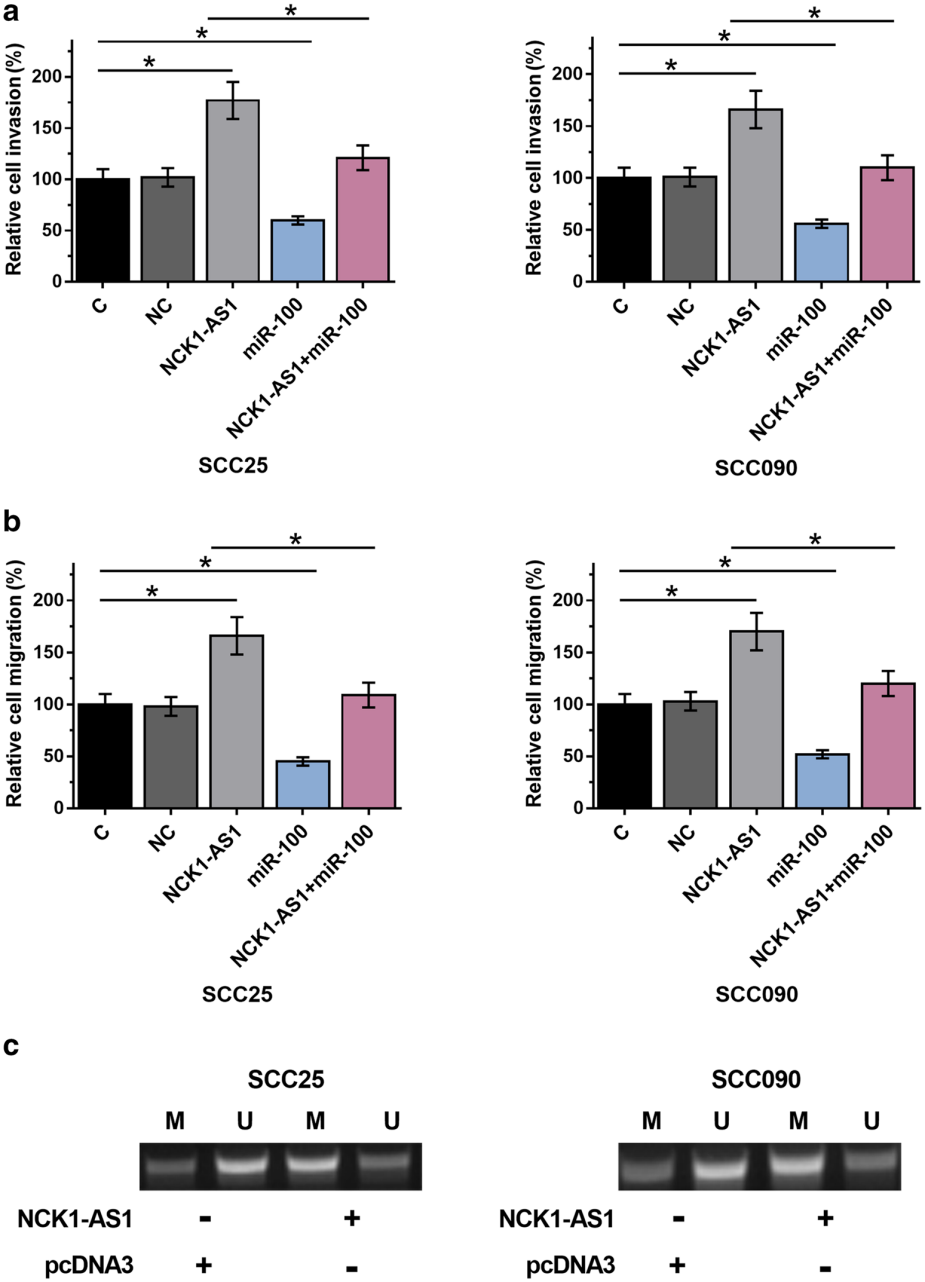
NCK1-AS1 promoted OSCC cell migration and invasion through miR-100. Transwell invasion and migration data analyzed by one-way ANOVA and Tukey test showed that overexpression of NCK1-AS1 resulted in a significantly increased rate of invasion (**a**) and migration (**b**) in comparison to C and NC groups. Overexpression of miR-100 played an opposite role and attenuated the effects of overexpression of NCK1-AS1 (*p < 0.05). MSP was performed to analyze the effects of overexpression of NCK1-AS1 on the methylation of miR-100 in both SCC25 and SCC090 cells. Experiments were repeated 3 times and representative images were presented (**c**)

Transwell invasion and migration assay revealed that, cells with the overexpression of NCK1-AS1 showed significantly increased rates of invasion (Fig. 5a) and migration (Fig. 5b) in comparison to that in the C group. Overexpression of miR-100 inhibited cell invasion and migration and attenuated the effects of overexpression of NCK1-AS1 (*p* < 0.05).

### Overexpression of NCK1-AS1 resulted in increased methylation of miR-100 in OSCC cells

MSP experiment showed that, compared to cells transfected with empty pcDNA3 vector, cells transfected with NCK1-AS1 expression vector showed obviously increased methylation (Fig. 5c).

## Discussion

The expression pattern, function and clinical values of NCK1-AS1 for OSCC have been investigated in this study. We found that NCK1-AS1 was upregulated in plasma of OSCC patients and upregulated expression of NCK1-AS1 showed early diagnostic values. In addition, NCK1-AS1 may promote the migration and invasion of OSCC cells by downregulating miR-100, which is downregulated in OSCC [12].

OU is a common reason for patients attending out-patient clinics. OSCC shares similar clinical symptoms to OU [13]. In some cases, OSCC can be misdiagnosed as OU, leading to delayed treatment. It has been well established that lncRNAs are critical players in OSCC and some lncRNAs have been demonstrated to be potential diagnostic and prognostic biomarkers for OSCC [14]. In this study we detected the expression of NCK1-AS1 in plasma of participants, indicating the existence of circulating NCK1-AS1 in blood. With the advantages of non-invasive nature, circulating lncRNAs have shown promising potentials in disease diagnosis [15]. We found that NCK1-AS1 was overexpressed in early-stage OSCC patients but not in OU patients. In addition, increased expression levels of NCK1-AS1 distinguished early-stage OSCC patients from OU patients and healthy participants. Therefore, NCK1-AS1 could possibly be used to diagnose early stage OSCC.

miR-100 participates in diverse biological processes, such as the regulation of breast cancer cell apoptosis and the regulation of lung cancer cell migration and invasion [16, 17]. In a recent study, miR-100 was found to be downregulated in OSCC and downregulation of miR-100 is involved in the development of OSCC [12]. Consistent results were obtained in this study.

We showed that NCK1-AS1 inhibited the expression of miR-100 in OSCC cells, while the mechanism is unclear. NCK1-AS1 was identified recently and our knowledge on its functions is limited. Interestingly, NCK1-AS1 has been proved to be a key regulator of cell proliferation and cell cycle progression [18]. This study proved that NCK1-AS1 can also regulate cancer cell migration and invasion. It has been reported that miR-100 can target FZD-8 and inhibit the Wnt/β-catenin signaling to inhibit the invasion and migration of breast cancer cells [19]. Moreover, miR-100 suppresses the invasion and migration by targeting IGF1R [20]. Therefore, NCK1-AS1 may regulate the expression of miR-100 to indirectly regulate the invasion and migration of OSCC cells. However, more studies are needed to identify the downstream effectors of NCK1-AS1 and miR-100 in this process. Our study enriched our knowledge of the function of this lncRNA. However, we did not include normal keratinocyte cells as control. Our future studies will try to address this issue.

## Conclusions

In conclusion, NCK1-AS1 was upregulated in OSCC and its diagnostic value is suggested. NCK1-AS1 may downregulate miR-100 to promote the migration and invasion of OSCC cells.

## Supplementary information

**Additional file 1: Figure S1.** Silencing of NCK1-AS1 resulted in the upregulation of miR-100 in OSCC cells.

## Data Availability

The datasets used and/or analyzed during the current study are available from the corresponding author on reasonable request.

## Abbreviations

OSCC: Oral squamous cell carcinoma
OU: Oral ulcer
AUC: Area under the curve
lncRNAs: Non-coding RNAs
