# Supplementary figures and images for: LncRNA NCK1-AS1 in plasma distinguishes oral ulcer from early-stage oral squamous cell carcinoma
Source: J Biol Res (Thessalon). 2020 Nov 11;27:16. doi: 10.1186/s40709-020-00126-1 (PMC7656691; doi:10.1186/s40709-020-00126-1)

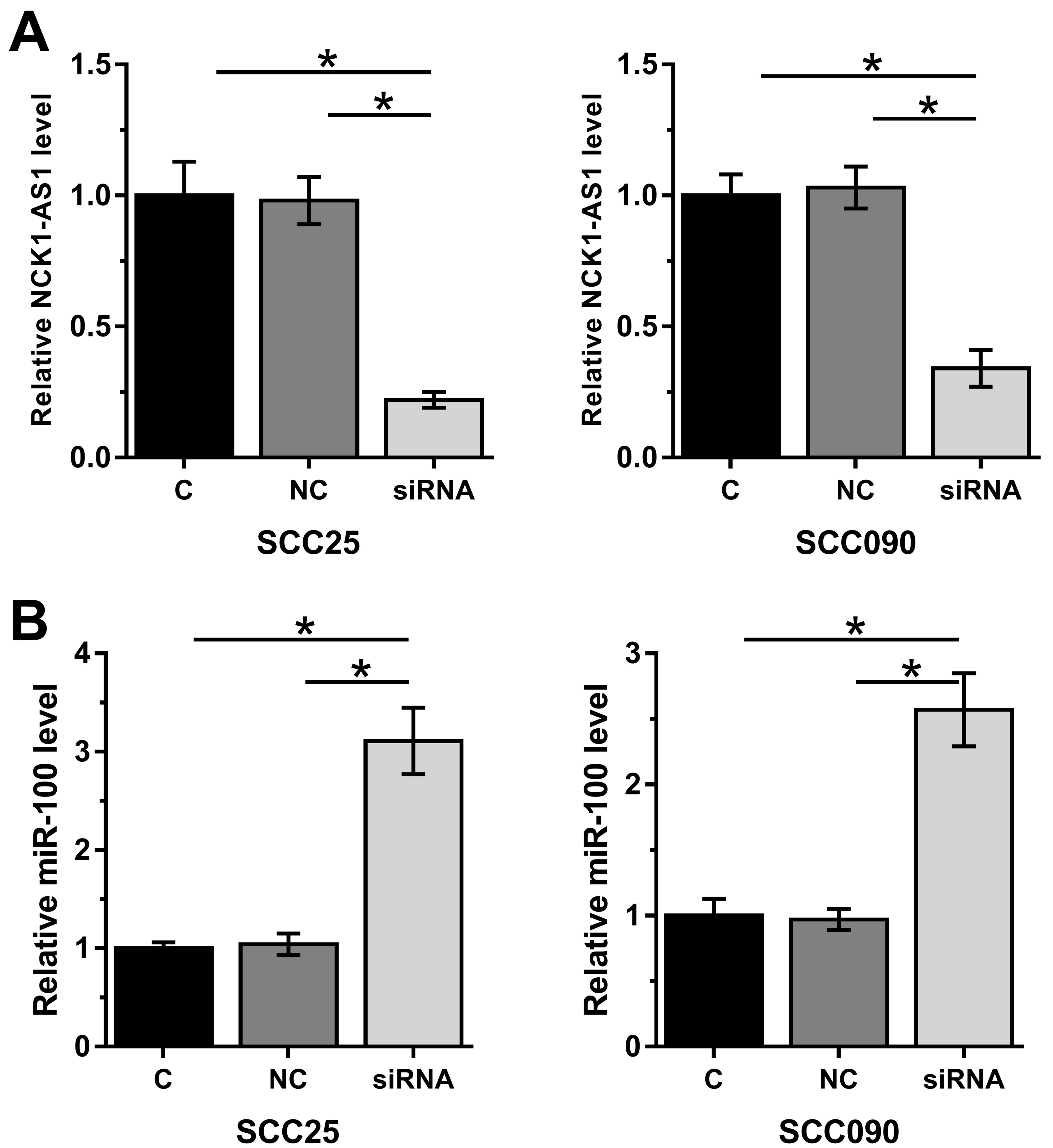

Supplement: Supplementary file 1 — Additional file 1: Figure S1. Silencing of NCK1-AS1 resulted in the upregulation of miR-100 in OSCC cells. [file 40709_2020_126_MOESM1_ESM.tif]
